# Supply kits for antenatal and childbirth care during antenatal care and delivery: a mixed-methods systematic review, the qualitative approach.

**DOI:** 10.1186/s12978-017-0299-0

**Published:** 2017-03-31

**Authors:** Mercedes Colomar, Maria Luisa Cafferata, Alicia Aleman, Giselle Tomasso, Ana Pilar Betran

**Affiliations:** 1 Montevideo Clinical Research Unit (UNICEM), Montevideo, Uruguay; 2 World Health Organization (WHO), Geneva, Switzerland

**Keywords:** Kits, Clean delivery kits, Maternal health, Systematic review, Qualitative studies

## Abstract

Antenatal care reduces maternal and perinatal mortality and morbidity through the detection and treatment of some conditions, but its coverage is less than optimal within certain populations. Supply kits for maternal health were designed to overcome barriers present when providing care during pregnancy and childbirth particularly to women from underserved population.

We conducted a mixed-methods systematic review on the use of supply kits. This manuscript presents the findings from qualitative studies that reported barriers, facilitators, and user’s recommendation in the adoption and implementation of any type of kit designed to be used during pregnancy or childbirth.

This review included eight studies, and seven were implemented in developing countries. Most studies assessed the implementation of clean delivery kits to be used during labour and delivery, and contributed to gain insights into factors that may hinder or foster the use of kits.

Clean delivery kits were conceived to cope with barriers related mainly to access. The most important barrier identified were those related to the socio-cultural and the lack of knowledge dimension such as who held the decision-making authority in the household, as well as popular beliefs behind the idea that birth preparation could bring bad luck, may prevent clients from adhering to their use. In addition, financial constraints and limited understanding of the instructions of use were accessibility barriers found. On the other hand, once used, clean delivery kits for maternal health were accepted by women and health workers. Convenience, hygienic components, and avoidance of delays in receiving care were viewed as satisfactory features.

Supply kits are mostly affordable and easily deployable. Increasing awareness among the population about the offered kits and providing information on their benefits emerges as a critical step to foster use in settings where kits are available. Implementation of this strategy requires low complexity resources and could make the use of kits an accepted alternative to increase the use of evidence-based interventions and thus improve quality of care during pregnancy, childbirth and neonatal period mainly at the community level in low income countries and remote areas with low access.

**Electronic supplementary material:**

The online version of this article (doi:10.1186/s12978-017-0299-0) contains supplementary material, which is available to authorized users.

## Plain English summary

Antenatal care improves maternal and perinatal health, but it has low coverage within certain populations. The supply kits for maternal health overcomes barriers when providing care during pregnancy and childbirth in underserved populations. We conducted a systematic review on the use of supply kits. We included eight studies, seven studies were from developing countries. Most studies assessed the implementation of clean delivery kits to be used during labour and delivery.

Kits were conceived to cope with barriers related to access. The most important barriers were socio-cultural, financial and related to lack of knowledge. Clean delivery kits for maternal health were accepted because they are convenient, hygienic, and avoid delays in receiving care.

Supply kits are affordable and easily deployable. This strategy can increase the use of evidence-based interventions and improve quality of care during pregnancy, childbirth and neonatal period mainly in areas with low access.

## Background

Most maternal deaths are preventable, as the evidence-based interventions to prevent or manage potential complications are well known. All women need access to antenatal care (ANC) during pregnancy, skilled care during childbirth, and care and support in the weeks after childbirth [1].

ANC reduces maternal and perinatal mortality and morbidity through the detection and treatment of conditions (pregnancy-related or not) that can increase the risk of adverse maternal and perinatal outcomes. Potential benefits of ANC are most significant in low-resources settings where mortality and morbidity levels among pregnant women and their neonates are highest [2, 3].

The Countdown 2015 tracked progress and remaining gaps in the 75 countries that accounts for more than 95% of all maternal, newborn and child deaths. In these countries the median coverage level of four or more ANC visits was 55% (range in coverage is 15 to 95%) [4–6].

The factors responsible for the low levels of coverage of ANC and the low quality of the care area multiple. For example, in Mozambique, chronic supply chain deficiencies, failures in the continuing education system, lack of regular audits and supervision and poor environmental conditions at the health center have been reported as factors that hinder the implementation of ANC and quality of care [7]. In addition, health care providers may have limited awareness of current clinical guidelines and a resistant attitude to adopting new recommendations, which limits the implementation of evidence-based care [7]. A systematic review found that maternal education, husband’s education, marital status, availability and cost related to ANC, household income, women’s employment, media exposure and having a history of obstetric complications were all factors affecting ANC [8]. Cultural beliefs and ideas about pregnancy also influenced ANC use in the same review. Parity had a statistically significant negative effect on adequate attendance. Whilst women of higher parity tend to use ANC less, there is interaction with women’s age and religion [8].

Many women are estimated to suffer pregnancy-related illnesses (9.5 million), near-miss events which are the life-threatening complications that women survive (1.4 million), and other potentially devastating consequences after birth [9, 10] Pregnancy-related illnesses and complications during pregnancy and delivery are associated with a significant impact on the foetus, resulting in poor pregnancy outcomes for both the mother and newborn [11]. Every year an estimated 60 million women give birth outside health facilities, mainly at home, and 52 million births occur without a skilled birth attendant [12].

A set of interventions provided to women and children during childbirth, can prevent at full coverage, 41 to 72% of newborn deaths, like clean and skilled care at delivery, newborn resuscitation, prevention of hypothermia, exclusive breastfeeding, clean umbilical cord care, and management of pneumonia and sepsis. A significant proportion of these mortalities and morbidities could be potentially addressed at the community level [13].

The experience of pilot programmes before the Alma-Ata Declaration, and subsequent trial evidence, suggests that community mobilization can bring about cost-effective and substantial reductions in mortality and improvements in the health of newborn infants, children, and mothers in a setting and strong community-based approaches. Although maternal survival requires improvements in comprehensive and basic obstetric care at hospitals and health centres, community mobilisation has an important role in improving care practices, increasing the use of safer motherhood services, promoting timely referral when problems arise, and reducing social disadvantage [14].

There is a current and critical need for approaches with potential for improving the uptake of interventions that have been proved efficient and beneficial in maternal health, specifically during pregnancy and childbirth. Developing countries and specially, low-resource settings, are particularly vulnerable to this lack of evidence. Supply kits (medicines and supplies packaged together for a specific healthcare task) have been proposed as a simple and low-cost solution to overcome some of the barriers to providing ANC and childbirth care to women from underserved population.

By the means of a systematic review, this manuscripts aimed to answer the following question; “what are the barriers and facilitating factors that affect the implementation of the use of supply kits for ANC and for childbirth care?

## Methods

We conducted a mixed methods systematic review of the literature in order to gather, describe and summarize worldwide experiences of users of supply kits for maternal care. This manuscript reports the findings of the review from studies which had a qualitative approach and methodology. The findings derived from the quantitative approach of this review are published somewhere else [15].

### Inclusion criteria

- Type of study designs
  For the qualitative component, we included studies that reported barriers, facilitators, and user’s recommendation in the adoption and implementation of any type of kit designed to be used during pregnancy or childbirth regardless of their content or the interventions for which they are intended.
- Type of kits
  Any type of kit designed to be used during pregnancy or childbirth was eligible for inclusion regardless of its content. For the purpose of this systematic review, kits were defined as a collection of medicines, supplies or instruments packaged together with the aim of conducting a healthcare task (e.g. antenatal care kit, caesarean section kit, clean delivery kit).
- Type of participants
  We included studies focusing on the perspectives of those who had experience with the implementation of kits (i.e. women, relatives, traditional birth attendants, doctors).
- Type of outcomes
  We included primary studies reporting participants’ viewpoints regarding the facilitating or impeding factors for the use, uptake and implementation of the supply kits.

### Search strategy

We conducted a broad search of the evidence taking into account the implementation of “kits for maternal health”. The terms for the search strategy for Pubmed included: Medical Supplies, Clean, Sanitary, Disposable Equipment, Kit, Birth Kit, Toolkit, Package, Box, Prenatal Care, Antenatal Care, Pregnancy Complications, Pregnancy, Postpartum Period, Labor, Obstetric, Intrapartum, Partum, Peripartum, Childbirth (Additional file 1: Annex I). The following additional electronic reference sources were searched: Cochrane Pregnancy and Child birth Group’s Trials Register, Campbell Collaboration, Lilacs and Embase. Other sites to identify unpublished studies were also searched (Additional file 1: Annex I).

No limits regarding publication date and no language restrictions were applied. For the purpose of this paper, only qualitative studies were considered.

### Study selection process

Citations identified through the search strategy in the electronic databases were imported in Early Review Organizing Software (EROS) and duplicates were deleted. EROS is a web-based software designed specifically to help in the performance of the first stages of systematic reviews by organizing citation distribution among authors, distributing the workload, facilitating independent revision of references, identification of duplicates, resolution of discrepancies, and incorporating quality assessment [16].

Two reviewers independently assessed the studies at each stage. In the first stage, all identified citations imported in EROS were screened based on title and abstract to select potentially relevant studies for full-text evaluation. In the second stage, full-texts of all selected citations were retrieved and assessed. Those fulfilling the inclusion criteria were included in the review.

The third stage included quality assessment of selected studies. Quality was assessed with a tool proposed by Mays & Pope, which includes assessing clarity of the research question, appropriateness of the design to answer the question, the context, sampling, and data collection and analysis procedures [17].

Data were extracted for the included studies using a data-extraction form specially designed for this review by the authors. The findings of each study were combined into a whole listing of themes, which described the phenomenon. Reviewers inferred barriers and facilitators from the views participants gave about the use of kits in general, captured by the descriptive themes, A thematic synthesis of the findings was conducted [18]. Reviewers translated themes and concepts from one situation to another, checking that each transfer was valid. We attempted to preserve context by providing structured summaries of each study detailing: aims, methods and methodological quality, and settings and samples. Results were presented in terms of barriers and facilitators for the use of kits for maternal health, and the elements were grouped according to the model of health service coverage and bottlenecks proposed by Tanahashi et al. [19].

This review was registered in Prospero Centre for Reviews and Dissemination, University of York with the number CRD42016043145.

## Findings

### Results of the search

**Fig. 1:**
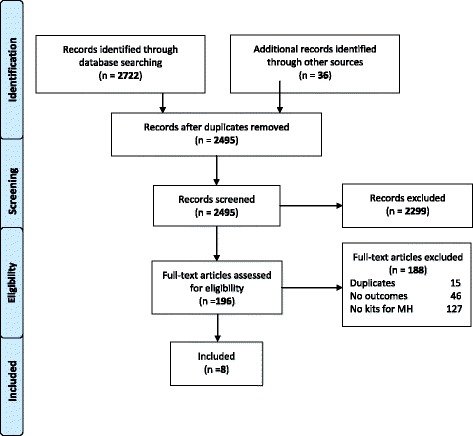
Flowchart Diagram

The search strategy identified 2495 unique citations. After assessing titles and abstracts for inclusion criteria, 2299 were excluded and after full text evaluation, 188 additional citations were excluded. Finally, eight citations were included in this qualitative approach of the review that reported experiences, barriers and facilitators for the implementation of the kits. Figure 1 shows the flowchart of this systematic review.

The eight included studies were published between 1992 and 2015. Seven studies were conducted in developing countries: three in Asia (Bangladesh and two in Nepal) and four in Africa (Kenya, Lesotho, Tanzania and Uganda) while one was conducted in the United Kingdom [20]. Seven studies focused on the use of kits during childbirth [20–26], and in one study the kits included commodities to be used during ANC, childbirth and early postnatal care [27]. The content of the kits of each study is summarized in Additional file 2: Annex II. The most common items in the kits were a cord tie/clamp and a clean plastic drape (6/8) followed by soap and sterile razor (5/8). Three kits contained gauze/cotton and two provided gloves.

Most of the studies assessed different aspects of the implementation of a so-called Clean Delivery Kit (CDK) conducted in African and Asian countries. Six studies [21–26] involving women from communities and birth attendants with and without training, explored acceptability of use and perceptions regarding CDK. The other two studies assessed the implementation of kits during antenatal care (study of homeopathic remedies in UK), delivery and post-natal care (co-packaged medicines to prevent transmission of HIV from mother to child in Lesotho).

Table 1 presents a summary of barriers and facilitators of the eight studies included in the qualitative analysis. Morrison 2015 explored the reasons for low CDKs utilization, and described the community perceptions of several types of CDKs in Nepal. They conducted 18 focus group discussions and 40 interviews with CDK users and non-users, service providers, birth attendants, and household decision makers in six districts. Interviews with central level personnel were also conducted. Lack of promotion and awareness about CDKs´ benefits; men or mother-in-laws having the decision-making prerogative, birth preparedness perceived as unnecessary because of the uncertainty of the birth outcome and, for some, the cost of the kits were the main barriers identified. On the other hand, reason for a positive regard of the kits were the convenience, hygienic components, and avoidance of delays in receiving care. The use of kits was also perceived to help the reputation of the TBAs by preventing illness.

**Table 1:** Systematized information of the barriers and facilitating factors for the use of the implementation of the kits

Author Year	Country	Objectives	Barriers	Facilitating factors
Kits during delivery - Clean Delivery Kits				
Morrison, 2015 [21]	Nepal	To explore the reasons for low CDKs utilization, and to describe community perceptions of CDKs in Nepal	*Acceptability*: - Cultural: CDKs users did not always hold the decision-making power to purchase or use the CDK. The decision to use the CDK was often made by the mother-in-law or husband of the pregnant woman, who might have limited knowledge of the CDK, or do not perceive its usefulness. - Popular Beliefs: Preparation might also be constrained by traditional belief that it would bring bad luck. *Accessibility*: - Financial: Although most people found the CDK price reasonable, some perceived it to be too expensive for poor people. - Health literacy: The use and recognition of CDKs was low among the families. *Contact*: - Lack of awareness: CDK potential benefits were not perceived (i.e. infection). *Availability*: - For some, CDKs was not easily available. Inadequate promotion.	*Acceptability*: CDK was generally well regarded by its users. The convenience and the hygienic components were the main reasons people said they used the CDK. Reputation - TBAs in particular felt that the CDK helped them maintain their professional reputation by preventing illness during births. Readiness to change - TBAs’ attitude toward the CDK as opinion leader was decisive in their promotional efforts. *Contact:* CDKs were perceived as “clean and safe” *Availability:* CDKs with all the necessary materials in one place might reduce delays in receiving care.
Dietsch, 2011 [22]	Kenya	To learn lessons from a traditional midwifery workforce in Western Kenya	Not mentioned	*Acceptability*; CDK were highly valued by TBA and might be an attraction to get linked with NGOs who distributed them and deliver seminars.
Waiswa, 2008 [23]	Uganda	To explore the acceptability and barriers to the recommended evidence-based practices. CDKs were one of the practices assessed as an evidence based intervention.	*Acceptability*: - Cultural: Decision making was a male prerogative - Popular Beliefs: Fear of preparing for the unborn whose viability is considered uncertain. *Accessibility:* - Out of stock in health units. - Financial*:* CDK were perceived as expensive.	*Contact:* Sanitary reasons: Using a new razorblade was considered important.
Winani, 2005 [24]	Tanzania	To gather information from CDK users and non-users in the community on the acceptability, correct use, and appropriateness of single-use, CDK.	*Accessibility*: - Health literacy: Misunderstanding of the pictorial instructions.	*Accessibility:* - Comprehension reasons: Even though the pictorial instructions were not well understood, women managed to use the CDK with no major complications. *Contact:* CDK were perceived as contributing to a clean delivery. Users showed willingness to pay for it, and they recommended it should` be used by other women.
PATH, 2002 [25]	Nepal	To understand the context of CDK use and non-use by women for their own childbirth and by women assisting them during delivery.	*Acceptability:* - Popular beliefs: Birth preparedness (BP) was a bad presage.- The highly ritual value of washing hands could inhibit real understanding of the need for washing hands (performed as ritual more than to reduce infection) - Cultural: Mothers themselves were not supposed to be involved in any birth preparation. Mothers had low decision-making autonomy. *Accessibility:* - Financial: Households often were not willing or able to spend the money to buy the CDK . - Health literacy: There was low understanding of the pictorial instructions. - The women expected to receive the CDK for free. In terms of how to spend the money, other materials and activities (name given ceremony) were seen as more important and get priority over the CDK. - - Also, most women did not know where to buy the CDK *Contact*: - Lack of Awareness: Weak perception of the usefulness of the CDK	*Acceptability:* The CDK was generally well regarded by its users. TBAs feel that the CDK helped them maintain their professional reputation by preventing illness during births. *Contact:* People were aware that dust and dirt might cause disease and that the CDK are clean and hygienic *Availability:* - Pragmatic: All supplies were available and ready to use in one place.
Nessa, 1992 [26]	Bangladesh	To produce a CDK that would appeal to potential buyers	*Accessibility:* - Health literacy: Misunderstanding of the pictorial message.	*Acceptability:* Users generally approved the CDK (The one that was produced with their inputs)
Kit for Antenatal Care, Delivery and Post natal care				
Steen, 2007 [20]	United Kingdom	To explore women’s experiences of using a self-administered kit of homeopathic remedies during the latter part of pregnancy, birth experience and the early postnatal period	Not mentioned	*Acceptability:* Women felt the kit could help them rather than feeling helpless or powerless. Many women and their partners expressed feelings of empowerment; the kit gave a focus.
McDougal, 2012 [28]	Lesotho	To examine the availability, feasibility, acceptability and possible negative consequences of a Minimum PMTCT Package, and to identify key learning from Lesotho’s experience with the Minimum PMTCT Package to inform future programming and evaluation of co-packaged medicines.	Accessibility: - Comprehension: Lack of patients’ ability to follow the complex instructions. Impatience of staff explaining patients. Contact: Providers expressed concern about adherence issues in women who had not disclosed their HIV status to their partners. Women might interrupt ANC and not deliver in facilities when they already obtain the medication in the Kit. Availability: Drug availability was a major bottleneck to scale up the implementation of the kit.	Acceptability: Providers seemed to have a positive attitude toward the Minimum PMTCT Package. They felt it saved lives and that it was reducing the number of babies born HIV positive. It could be rapidly scaled up; it was feasible to deliver within the context of routine ANC; it was acceptable to most of providers and clients; it did not appear to adversely affect the quality of ANC; and it did not affect the care among exposed infants in the first months of life. Availability: Most women were very happy to have medication to prevent their child from getting sick. Providers perceived it as a potentially life-saving intervention

Dietsch 2011 aimed to describe the experience and the lessons learned from the TBAs workforce in Kenya using CDKs. Interviews were conducted with 84 participants. The kits contained soap, gloves, clean plastic drape, cord tie/clamp and gauze/cotton (see Additional file 2: Annex II). Kits were highly valued by TBAs and often attracted them to assist to seminars taught by the NGO that distributed the CDKs. These seminars often focus on risk assessment, prevention and management of obstetric emergencies as well as effective use of the birth kits.

In Uganda, Waiswa 2008 aimed to explore the acceptability and barriers to the recommended evidence-based practices and to home-visiting by a community volunteers. The so-called *mamma kit* in this study was a CDK, and it was one of the interventions assessed as part of the evidence based recommendations. Barriers reported were related to accessibility issues (out-of-stock in health units), and cost of the kits. Acceptability was related to cultural reasons such as the male having the prerogative of decision-making and the fear of preparing for what is considered an uncertain event in settings where negative perinatal outcomes, were frequently reported.

In Tanzania, Winani 2005 implemented a study to gather qualitative information from kit users and kit non-users in the community on the acceptability, correct use, and appropriateness of the kits. In-depth interviews were conducted among a random sample of kit users and non-users across clusters. The kit items were packaged together in a sealed plastic bag. Lack of clarity and misunderstanding of the pictorial instructions were identified as main barriers. The association of the kits with cleanness and a positive appraisal from kit users were facilitators for their use in this study.

In Nepal where the majority of births occur at home and are attended by people with little or no training, shortages of suitable clean materials contribute to the problem of perinatal infection. To address this problem, a disposable CDK was produced. PATH in 2002 aimed to enhance the understanding of the context of use and non-use of CDK by women, for their own childbirth and by women assisting them during delivery. Kit users, non-users, trained and untrained birth attendants and family attendant were interviewed. The kits contained a clean plastic drape, a sterile razor; a cord tie/clamp and a plastic coin (see Additional file 2: Annex II). As found in Uganda [22], popular beliefs and cultural issues were the main barriers identified, jointly with lack of perception of the usefulness of the kit and financial constraints which lead to lack of priority of the kit over the acquisition of other materials or activities related to the birth.

The implementation of a CDK, was a strategy to overcome the challenge of maternal and neonatal infection in Bangladesh. In 1992, Nessa aimed to produce a kit that would appeal to potential buyers and could be made available at low price. Potential users were interviewed and the content of the kit was a compromise between what was feasible for the price and what potential buyers requested. The kits were tested and women generally approved them. As in Tanzania, the major barrier was the misunderstanding of the pictorial instructions.

The only study conducted in a high-income country [20] assessed the use of a self-administered kit of homeopathic remedies during later childbirth in women in the United Kingdom. One of the study objectives was to increase women’s knowledge of homeopathy and to keep the self-administration simple by using brief descriptions of which remedies may be helpful to alleviate physiological and emotional disturbances which occur commonly during early labor. The study explored women’s experiences and reported feelings of empowerment (see Table 1).

McDougal in 2012 evaluated the implementation of kits for ANC, childbirth and postnatal care in Lesotho. It aimed to examine the availability, feasibility, acceptability and possible negative consequences of the *Minimum Prevention to Mother to Child Transmission (PMTCT) Take-away Package* during ANC. A package containing all necessary antiretroviral (ARV) medications for pregnancy were distributed at the first ANC visit. If the first ANC visit occurred beyond 28 weeks gestation, women were supplied with a month of ARV prophylaxis until results of testing were obtained. The study used a qualitative and quantitative approach, and providers and clients were interviewed. They reported concerns about the complexity of instructions, adherence issues particularly in women who do not disclose their HIV status and the counterproductive effect of interrupting ANC in women who already obtained the needed medication in the kit (Table 1). On the other hand, the kits developed a positive attitude among health care providers and women with a clear understanding of the health benefits to prevent HIV transmission.

### Quality of evidence

A summary of methodological quality assessment is presented for each domain (relevance, data collection and analysis, sampling, bias assessment, appropriate design to answer the research question, context description, and clarity of the research question) (Additional file 3: Annex III).

In all studies, the research question was clear and designs were appropriate, contexts or settings were adequately described, with systematic data collection and data analysis in 7 of the 8 studies. The sampling was appropriate in six of the eight studies. In all studies, the risk of bias was assessed and findings reported contributed usefully to knowledge.

## Discussion

### Main findings

This systematic review identified eight studies that contributed to gain insights into factors that may hinder or foster the use of supply kits for care during pregnancy, labor and childbirth. Six studies reported on the use of kits for childbirth and two of them, included also components for antenatal care. The findings from these qualitative studies showed that kits were conceived and designed to cope with barriers related mainly to access. All but one were studies conducted in developing countries from Asia and Africa, in settings where the majority of women give birth at home alone, or with the assistance of a TBA. It is in these setting where kits may maximize its impact on maternal and newborn health.

The results were uniform across studies pointing to the same issues and difficulties in all settings. Tanahashi in 1978 [19] proposed a four-dimension model for access to health care. These dimensions were: acceptability (cultural inconsistency between users and health professionals, myths, social stigma, distrust of health professional), accessibility (price, distances, waiting times), contact (lack of knowledge, lack of perception of a health problem), and availability (lack of resources and information). Applying this model to our findings, the most important barriers identified were those related to the socio-cultural and the lack of knowledge dimension (acceptability). Regarding specifically the implementation of CDK, the interaction of poverty, ignorance or unawareness of potential benefits, carelessness, and women disempowerment, was suggested to prevent some families from preparing for birth and acquiring a kit. In African and Asian countries, women give birth at home following traditional practices that are often harmful, and have misconceptions related to the cause of infections. Issues related to the cultural tradition such as who holds the decision-making authority, and popular beliefs, such as that birth preparation could bring bad luck, may prevent clients from adhering to their use. In addition, financial constraints and limited understanding of the instructions of use were some of the accessibility barriers found. On the other hand, among users, satisfaction leading to approval of the CDKs was reported and the perception of kits’ benefits for their own health was an asset to foster use. In addition, for TBAs, the easy and quick access to the supplies was emphasized as a time saving factor promoting utilization among them. TBAs highlighted that the kit actually prevents illness during birth and this fact, would help them maintain their professional reputation.

The results of this review point to empowerment, education and promotion as a critical component to increase the use of the kits during pregnancy, labor and childbirth. The entire family, including mother-in-law, but also TBAs, should be targeted as influential facilitators to scale up the implementation of use of kits, while gaining skills, confidence and control over childbirth would directly empower women [28]. Once users are exposed to the kits, they seem to appreciate these benefits and seem to value receiving everything ready to use in one single pack, which is viewed as a satisfactory strategy to reduce the delay in receiving care.

Factors pointed by this review affecting the use of the CDK are not very different from barriers and facilitators found in other studies assessing the utilization of other health services within ANC [7, 29, 30]. In several African and Asian countries, it is a common practice among pregnant women to seek for traditional medication or TBAs as first line providers since they perceive facility birth as less convenient. There is lack of reliable transportation, childbirth plans are not acceptable, health care expenses are not affordable, and they perceived negative attitudes from health professionals [31, 32]. This indicates that even very specific interventions or tools, such as supply kits, designed to overcome health system limitations in low-resource settings, need to consider in its implementation the very same factors that affects the utilization of other health services.

Only one study was performed in a high-income country. The objective of this study was not to reduce maternal or neonatal mortality on morbidity, but the authors were also aligned with the idea of promoting autonomy and the empowerment in women, like in the other studies assessing kits for the promotion of maternal and neonatal health [20].

The use of kits is intended to facilitate the provision of interventions, by delivering in one shot all the elements a woman needs in a given situation during ANC, delivery or postpartum. In addition, it optimizes the scarce contacts with a target population in low resource settings where barriers related to accessibility, cultural aspects, knowledge and lack of satisfaction with the services; prevent women from engaging to ANC. The successful implementation of kits minimizes the burden of acquiring each of those goods separately, what implies having the knowledge of what it is needed and how it is supposed to be used, and it saves time in acquiring them. This is the main reason why kits for maternal health (CDK & kits for PMTCT) are usually implemented in low-income countries, where maternal and neonatal mortality remains high.

### Strength and limitations

This review has several strengths. We developed a broad and comprehensive search strategy including published and not published manuscripts and documents. We included studies with a variety of methodological approaches with different views of the same problem. A standardized methodology for assessment of the quality allowed determining strength and limitation of the primary studies. This is the first published systematic review that provides a comprehensive overview of the background on the use of kits, the barriers for its implementation and factors that foster its utilization, as well as the effectiveness of the strategy, which is published elsewhere [15].

This review has some limitations. The results described can only be generalized to low income settings, since the use of kits have been proven effective and acceptable among deprived pregnant women. Most studies reporting implementation of kits for maternal health were specific CDK designed to be used during childbirth and no specific conclusions can be drawn about the use of kits during pregnancy. Limitation of this review are related to the heterogeneity of the definition of kits and components and the quality of the studies, hindering full comparability.

## Conclusions

Supply kits for maternal health are accepted by women and health workers. CDK specifically are mostly affordable and easily deployable. Increasing awareness among the population about the offered kits and providing information on their benefits emerges as a critical step to foster use in settings where kits are available. Implementation of this strategy requires low complexity resources and impact could be probably large what makes the use of kits an accepted alternative to increase the use of evidence-based interventions and thus improve quality of care during pregnancy, childbirth and neonatal period, at the community level, in low-income countries and remote areas with low access.

## Additional files

Additional file 1: Annex I Search Strategy. (DOCX 15 kb)

Additional file 2: Annex II Components of the kits. (DOCX 18 kb)

Additional file 3: Annex III Quality assessment by individual study and summary of methodological quality assessment of risk of bias. (DOCX 94 kb)

## Abbreviations

ANC: Antenatal Care
CDK: Clean Delivery Kits
EROS: Early Review Organizing Software
NGO: Nongovernmental Organization
PATH: Program for Appropriate Technology in Health
TBA: Traditional Birth Attendants
