# Supplementary material for: Supply kits for antenatal and childbirth care during antenatal care and delivery: a mixed-methods systematic review, the qualitative approach
Source: Reprod Health. 2017 Mar 31;14:48. doi: 10.1186/s12978-017-0299-0 (PMC5374621; doi:10.1186/s12978-017-0299-0)
Supplement: Supplementary file 1 — Annex I Search Strategy. (DOCX 15 kb) [file 12978_2017_299_MOESM1_ESM.docx]

**Annex I.**

**Search Strategy**

(((Medical Supplies[tiab] OR Clean[tiab] OR Sanitary[tiab]) AND (Disposable Equipment[tiab] OR Kit[tiab] OR Kits[tiab])) OR birth kit*[tiab] OR birth kits[tiab] OR Birth toolkit*[tiab] OR Birth Package*[tiab] OR Medical Kit*[tiab] OR Medical Toolkit*[tiab] OR Medical Package*[tiab] OR Intervention Kit*[tiab] OR Intervention Toolkit*[tiab] OR Intervention Package*[tiab] OR Intervention Supplies[tiab] OR Interventional Supplies[tiab] OR Medical Box*[tiab] OR Intervention box*[tiab] OR Interventional box*[tiab]) AND (Prenatal Care[Mesh] OR Prenatal Care*[tiab] OR Pre-Natal Care*[tiab] OR Antenatal Care*[tiab] OR Ante-Natal Care*[tiab] OR Pregnancy Complications[Mesh] OR Pregnancy[tiab] OR Postpartum Period[Mesh] OR Postpartum[tiab] OR Post Partum[tiab] OR Puerperium[tiab] OR Puerperal[tiab] OR Postnatal Care[Mesh] OR Post Natal[tiab] OR Postnatal[tiab] OR Labor, Obstetric[Mesh] OR Labor[tiab] OR Labour[tiab] OR Obstetric Labor Complications[Mesh] OR Intrapartum[tiab] OR Partum[tiab] OR Peripartum[tiab] OR Childbirth[tiab])

**Databases**

United Nations Population Fund (UNFPA), World Health Organization (WHO), Panamerican Health Organization (PAHO), Gates Foundation, World Bank, United Nations Children's Emergency Fund (UNICEF), Program for Appropriate Technology in Health (PATH), Prevention of Postpartum Hemorrhage Inititative (POPPHI), Safe Motherhood, Save the Children, Royal College of Obstetrics and Gynecology, National Institute for Health and Care Excellence (NICE), United Nations Aids (UNAIDS), WELLCOME TRUST, National Institutes of Health (NIH), Global Health Technologies Coalition, Johns Hopkins Program for International Education in Gynecology and Obstetrics (JHPIEGO), Population Council, International Maternal and Child Health Foundation, Care International, Society for Nutrition, Education & Health Action (SNEHA), Canadian Network for Maternal, Newborn and Child Health, Pathfinder International, Women Deliver, Women and Health Alliance International (WAHA), Family Care International, The Partnership for Maternal, Newborn and Child Health, Internation Federation for Gynecology and Obstetrics (FIGO), Federacion Latinoamericana de Sociedades de Obstetricia y Ginecologia (FLASOG), United States Agency for International Development (USAID), Birthing Kits Foundation Australia (BKFA), Australian Government's overseas Aids Programme (AusAID), Dona International, The Global Fund, Institute of Reproductive Health (IRH), Maternal Adolescent and Child Health System, John Snow Incorporation (JSI) Research & Training Institute, March of Dimes.
