# Supplementary material for: Supply kits for antenatal and childbirth care during antenatal care and delivery: a mixed-methods systematic review, the qualitative approach
Source: Reprod Health. 2017 Mar 31;14:48. doi: 10.1186/s12978-017-0299-0 (PMC5374621; doi:10.1186/s12978-017-0299-0)
Supplement: Supplementary file 2 — Annex II Components of the kits. (DOCX 18 kb) [file 12978_2017_299_MOESM2_ESM.docx]

**Annex II.**

**Components of the Kits**

|  | **Soap** | **Gloves** | **Clean plastic drape** | **Sterile razor** | **Cord tie/clamp** | **Gauze/cotton** | **Antiseptic** | **Newborn cap** | **Other** | **free** |
| --- | --- | --- | --- | --- | --- | --- | --- | --- | --- | --- |
| **Pregnancy and Labor** |  |  |  |  |  |  |  |  |  |  |
| Mc Dougal 2012 |  |  |  |  |  |  |  |  | HIV treatment |  |
| Steen 2007 |  |  |  |  |  |  |  |  | Homeopathic remedies |  |
| **Childbirth** |  |  |  |  |  |  |  |  |  |  |
| Winani 2005 | x |  | x | x | x |  |  |  | Pictorial instruction | free |
| Morrison 2015 | X |  | X | x | x |  |  |  | Pictorial instruction |  |
| PATH 2002 |  |  | x | x | x |  |  |  | plastic coin |  |
| Nessa 1992 | x |  | x | x | x | x |  |  | Pictorial instruction | Free/low price |
| Dietsch 2011 | x | x | x |  | x | x |  |  | Sterile scalpel |  |
| Waiswa 2008 | x | x | x | x | x | x | - | - | Maama kit | Low cost |
