# Supplementary material for: Supply kits for antenatal and childbirth care during antenatal care and delivery: a mixed-methods systematic review, the qualitative approach
Source: Reprod Health. 2017 Mar 31;14:48. doi: 10.1186/s12978-017-0299-0 (PMC5374621; doi:10.1186/s12978-017-0299-0)
Supplement: Supplementary file 3 — Annex III Quality assessment by individual study and summary of methodological quality assessment of risk of bias. (DOCX 94 kb) [file 12978_2017_299_MOESM3_ESM.docx]

**Methodological quality assessment**

| **ID** | **Clarity of the research question** | **Context** | **Appropriate design to answer the research question** | **Bias assessment** | **Sampling** | **Data collection and analysis** | **Relevance** |
| --- | --- | --- | --- | --- | --- | --- | --- |
| Dietsch 2010 | 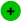 | 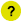 | 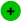 | 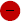 | 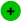 | 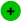 | 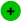 |
| McDougal 2012 | 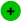 | 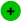 | 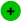 | 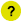 | N/A | 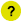 | 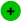 |
| Morrison 2015 | 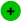 | 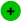 | 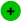 | 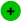 | 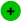 | 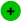 | 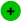 |
| Nessa 1992 | 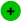 | 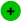 | 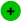 | 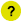 | 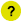 | 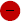 | 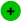 |
| PATH 2002 | 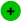 | 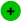 | 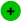 | 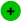 | 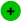 | 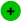 | 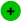 |
| Steen 2007 | 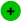 | 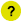 | 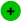 | 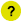 | 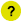 | 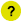 | 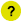 |
| Waiswa 2008 | 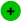 | 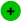 | 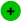 | 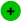 | 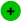 | 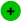 | 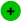 |
| Winani 2005 | 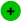 | 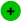 | 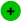 | 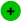 | 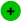 | 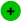 | 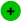 |
